# Previously treated versus untreated scoliosis: are results different?

**DOI:** 10.1186/1748-7161-9-S1-O21

**Published:** 2014-12-04

**Authors:** Fabio Zaina, Sabrina Donzelli, Monia Lusini, Salvatore Minnella, Stefano Negrini

**Affiliations:** 1 ISICO, Milan, Italy; 2 Don Gnocchi - Brescia University, Brescia, Italy

## Background

Every day we see patients coming after some kind of brace treatment for adolescent idiopathic scoliosis (AIS). Literature is made mainly by studies about untreated patients, so we don’t know what to expect when we start such a treatment.

## Aim

The aim of the present study was to compare the results of previously treated scoliosis compared to untreated ones.

## Design

Prospective observational controlled cohort study nested in a prospective database started in March 2003.

## Methods

Inclusion criteria: patients that started a brace treatment at their first clinical evaluation at our institute between 2003 and 2009 for AIS, 12-15 years old, Risser 0-3.

Patients were divided in two groups, one of patients already treated with a brace (BRACE Group), and one never treated before (UNTREATED Group).

## Outcome measure

The threshold of 5° Cobb to define worsened, improved and stabilized curves was considered, average Cobb angle, ATR, TRACE (for aesthetic evaluation) Statistical analyses: Mean and SD were used for descriptive statistics of clinical and radiographic changes. Relative Risk of failure (RR), 95% Confidence Interval (CI), Student’s t, Kruskall Wallis, and chi square test were applied.

## Results

268 patients were included (226 females), age 13.3 (±1).

BRACE Group: 108 (96 females), age 13.2 (±1), Cobb Angle 34±12°, ATR 9.6±0.4, TRACE 5.7.

UNTREATED Group: 160 (130 females), age 13.2 (±1), Cobb Angle 33±11° ATR 10.3±0.2°, TRACE 5.

No differences among groups at first visit but for TRACE (p<0.05).

49.38% of patients improved in UNTREATED, 43.13 stable, 7.50 worsened vs 35.19, 52.78 and 12.04 for BRACE (p=0.06). The Cobb angle was 28.9 vs 30.1 (p=0.06). The RR of failure for BRACE was 1.6 (IC95%0.86-2.35). No differences among groups for TRACE and ATR. Drop out had results similar to the completers (NS).

## Conclusions

Average clinical and radiological parameters improved in both groups. In the UNTREATED group results were slightly better even not significant, probably for the low statistical power. This study demonstrate that with a good treatment it’ possible to achieve good clinical results even in already treated patients.
